# Eggshell calcification after intrathyroidal hemorrhage of retrosternal thyroid

**DOI:** 10.1186/1757-1626-1-11

**Published:** 2008-05-25

**Authors:** Mehmet Fatih Yuzbasioglu, Mesut Ozkaya, Fikret Ezberci, Nimet Senoglu, Betul Kizildag

**Affiliations:** 1 Department of General Surgery, Kahramanmaras Sutcu Imam University, Medical Faculty, Kahramanmaras, Turkey; 2 Department of Endocrinology and Metabolism, Kahramanmaras Sutcu Imam University, Medical Faculty, Kahramanmaras, Turkey; 3 Department of Anesthesiology and Reanimation, Kahramanmaras Sutcu Imam University, Medical Faculty, Kahramanmaras, Turkey; 4 Department of Radiology, Kahramanmaras Sutcu Imam University, Medical Faculty, Kahramanmaras, Turkey

## Abstract

We report a rare event of old hemorrhage into a thyroid causing respiratory distress. A 67-year-old man with chronic cough and recent dysphagia was found to have a retrosternal mass extending into the visceral mediastinum on chest roentgenogram. A computed tomographic (CT) scan confirmed eggshell callcification, which was 53 × 53 × 80 mm in size a retrosternal thyroid mass and revealed significant tracheal deviation to the right due to an extensive mass surrounded by a calcificated capsule in the left lobe of the thyroid gland with extension to the upper mediastinum. He successfully underwent left lobectomy of the thyroid gland with sternotomy. The pathological examination revealed intrathyroidal hemorrhage of the thyroid gland with massive intracystic old bleeding.

## Introduction

Eggshell calcification is one of the three patterns of intrathyroidal calcification. Others are dystrophic calcification, and fine stippled psammomatous calcification [1]. Calcification within the thyroid gland is not uncommon, and it presence has been reported in up to 21% of plain X-rays [2]. Generally, eggshell calcification is seen in benign nature, but this type of calcification has been reported in one case report associated with follicular thyroid carcinoma [3].

Hemorrhage into the thyroid has been well reported in the literature but eggshell calcification because of old haemorrhage is rare. We presented an unususal eggshell calcification retrosternally on the chest roentgenogram.

## Case Presentation

**Figure 1:**
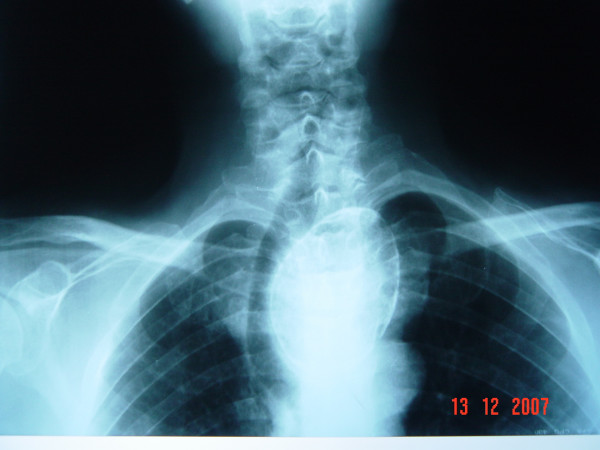
Chest roentgenogram demonstration of an eggshell calcificated mass.

**Figure 2:**
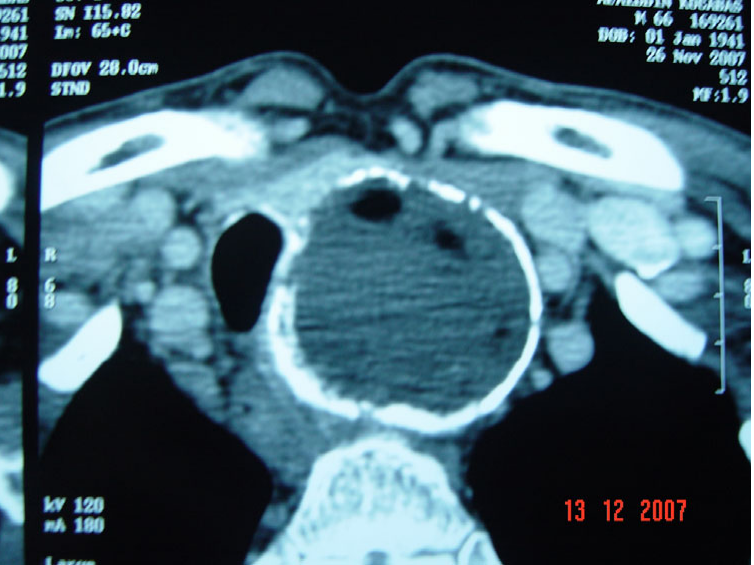
CT scan demonstration of a calcificated mass downside of the left lobe of the thyroid gland.

**Figure 3:**
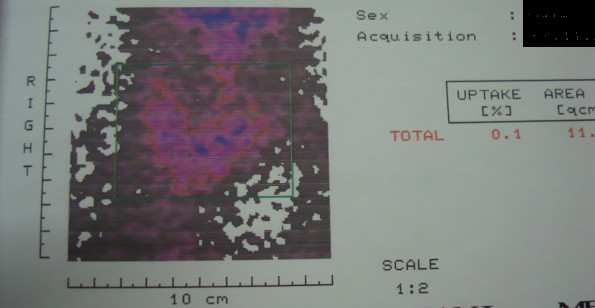
Tc-99m pertechnetate thyroid scan demonstrated a mass at the bottom of the left lobe in the thyroid gland with low uptake.

A 67-year-old male patient was investigated for nonproductive cough associated with dyspnea and recent dysphagia for solid foods. Clinical examination revealed tracheal deviation to the right side. Chest roentgenogram demonstrated an eggshell calcificated mass to the left of the midline in the upper mediastinum pushing the trachea rightward (Figure 1). Computed tomography (CT) scan with intravenous contrast demonstrated an extensive mass surrounded by a calcification downside of the left lobe of the thyroid gland with extension to the upper mediastinum and significant tracheal deviation to the right (Figure 2). The mass was 53 × 53 × 80 mm in size. Angio-CT demonstrated that no communicated vessel with the mass. Tc-99m pertechnetate thyroid scan demonstrated a mass at the bottom of the left lobe in the thyroid gland with low uptake (0.1%, normal range: 0.5% to 3.50%) corresponding to the radiological demonstrated mass (Figure 3). Because of low uptake in thyroid scan, it was reported as thyroiditis.

In his previous medical history, he had cerebral hemorrhagic stroke in year 2000. His physician prescribed acetylsalicylic acid 100 mg (Coraspin 100 mg, Bayer). After a gastrointestinal bleeding, he left using acetylsalicylic acid with his physician recommendation. But he was not aware of an acute swelling in thyroid region and acute respiratory distress at the gastrointestinal bleeding term.

The WBC was 8300/L (normal, 3500–9700); hemoglobin was 12.0 g/dL (normal, 11.2–15.2); platelet count was 241 × 10^3 ^K/uL (normal, 142 – 424 × 10^3^). Prothrombin time and partial thromboplastin time were 84% (normal, 80 – 120) and 26.9 second (normal, 26.0 – 38.0), respectively. Thyroid profile results demonstrated TSH of 1,25 μIU/mL (normal, 0.4 – 4.0); free T3, 2.18 pg/mL (normal, 1.8 – 4.3); free T4, 0.975 ng/mL (normal, 0.2 – 1.9); thyroglobulin in blood, 24 ng/mL (normal, 0 – 55).

**Figure 4:**
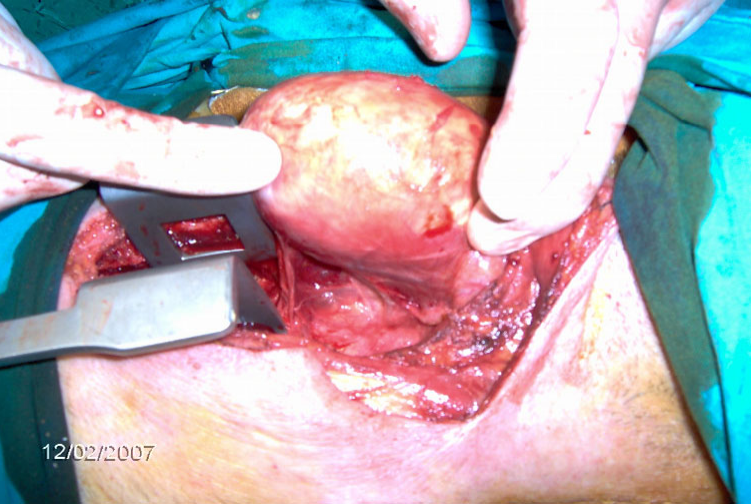
Left lobectomy with sternotomy.

**Figure 5:**
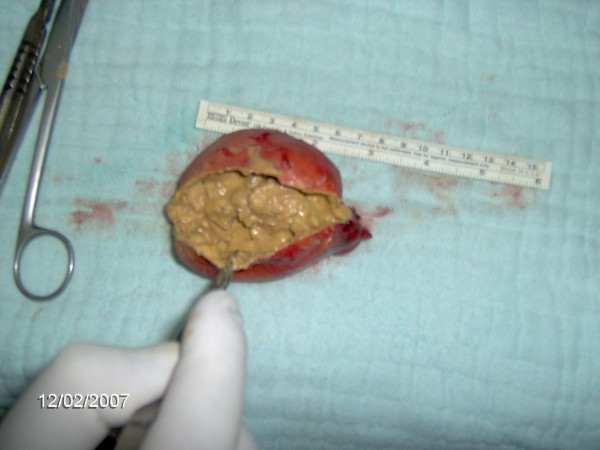
The cut surface of resected lobe revealed a distinct yellow- brown muddy content.

**Figure 6:**
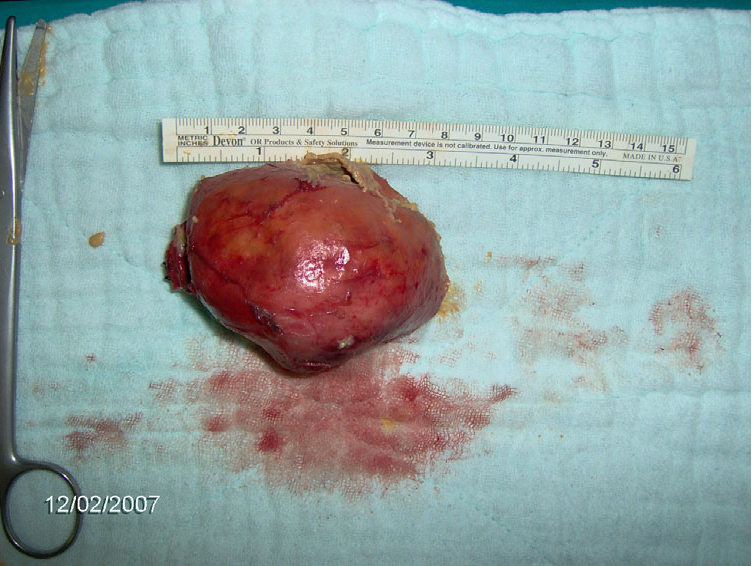
The calcification in the periphery.

He was taken to surgery for thyroid exploration. The operative findings were that the left lobe of thyroid gland was swollen, tense, and calcificated, expanding to the superior mediastinum, whereas the right lobe was normal. Left lobectomy was successfully performed with sternotomy (Figure 4). On gross examination, the cut surface of resected lobe revealed a distinct yellow- brown muddy content, 8 cm in greatest diameter, located in the whole of the left thyroid gland (Figure 5). A rim of the calcification in the periphery was apparent (Figure 6). The pathological examination revealed eggshell calcification of the thyroid gland with old massive intrathyroidal bleeding. He recovered uneventfully. He was discharged in good condition on the 3rd postoperative day. Three months after the operation, the thyroid function was normal.

## Discussion

First report of hemorrhage into the thyroid gland is described by Simon in 1894. To date reported cases about haemorrhage into thyroid was caused by trauma, cervical hyperflexion, a manual blow to the back of the head, lifting a heavy weight, straining at defecation, and even during household work [2]. And to date eggshell calcification in thyroid gland was reported but none of them was caused after intrathyroidal hemorrhage as in our case report.

This case is unusual in that eggshell calcification of intrathyroidal hemorrhage was located in the retrosternal, a finding not previously reported in the English literature. Ring-shaped calcifications may simulate the eggshell appearance. These include aneurysms of the great vessels, parathyroid tumors, pulmonary arteries in pulmonary arterial hypertension, thymic cysts, and thyroid tumors [4]. We had performed angio-CT for communications of vessel with the mass. Angio-CT was reported as no communications occured with the mass.

Bleeding is the most frequent complication of oral acetylsalicylic acid therapy. The most common locations for bleeding include gastrointestinal and genitourinary tracts. Platelet inhibition via acetylsalicylic acid can increase the risk of bleeding but it is used safely in cardiovascular diseases and stroke [5]. Our patient had no underlying coagulopathy except that he had acetylsalicylic acid usage for two years the gastrointestinal bleeding episode. It is possible, although unlikely, that a mild coagulopathy from aspirin ingestion predisposed him to this event. But in our case, we could not predict that acetylsalicylic acid usage was the main casuse of intrathyroidal bleeding.

Eggshell calcification of thyroid is rare and only three cases have been reported. One was an ultrasonic appearance of an eggshell calcification of a thyroid nodule reported in 1978 by Gooding GA [6], other was eggshell calcification in follicular thyroid carcinoma reported in 2005 by Cheng SP and et al [3]. The last and recent one was double eggshell calcification in thyroid in 2007 by Vandemergel X [7].

Egg-shell calcification is one of the patterns of dystrophic calcifications and is often associated with multinodular goiters [8]. It was generally thought to be an indicator of benignancy [8], however, cases of papillary carcinoma [4] and undifferentiated carcinoma [9] associated with this type of calcification have been reported. In Taki S et al series, 43% (6/14) of this type of calcification was associated with cancer, and all of them were papillary carcinoma [10].

Typical benign nodules are well defined, mostly cystic, and hyperechoic relative to adjacent parenchyma (96% benign). These nodules have eggshell calcification and a thin, echolucent halo around the entire lesion, and they always contain internal debris. Lesions demonstrating eggshell calcification and a thin echolucent halo around the entire lesion are most often benign. Some authors have found that the halo sign is present in 21–33% of thyroid cancers. But Cheng SP considered that type of thyroid calcification is not a good indicator of benignancy [3].

## Conclusion

In conclusion, this is the first report on an eggshell calcification in intrathyroidal hemorrhage with unknown etiology. The cause of the intrathyroidal hemorrhage could not firmly be delineated, although it remains possible that an unusual trauma triggered this rare medical condition.

## Consent

Written informed consent was obtained from the patient for publication of this case report. A copy of the written consent is available for review by the Editor-in-Chief of this journal.

## Authors' contributions

MFY carried out the operation, the design of the study and drafted the manuscript, FE participated in operation part, MO was involved the clinical preparation of the patient, NS conducted anesthesia of the operation, BK carried out the preparation of radiological findings. All authors read and approved the final manuscript.
